# Supplementary material for: Overlapping cell population expression profiling and regulatory inference in C. elegans
Source: BMC Genomics. 2016 Feb 29;17:159. doi: 10.1186/s12864-016-2482-z (PMC4772325; doi:10.1186/s12864-016-2482-z)
Supplement: Additional file 13: — Web supplement. (DOC 21 kb) [file 12864_2016_2482_MOESM13_ESM.zip › sortWeb/clusters/hier.300.clusters/56.html]

Cluster 56 

## Cluster 56

### Expression

| cnd-1 rep. 1 | cnd-1 rep. 2 | cnd-1 rep. 3 | pha-4 rep. 1 | pha-4 rep. 2 | pha-4 rep. 3 | ceh-27 | ceh-36 | ceh-6 | F21D5.9 | mir-57 | mls-2 | pal-1 | pros-1 | ttx-3 | unc-130 | hlh-16 | irx-1 | ceh-6 (+) hlh-16 (+) | ceh-6 (+) hlh-16 (-) | ceh-6 (-) hlh-16 (+) | cnd-1 singlets | pha-4 singlets | 0 | 60 | 120 | 150 | 180 | 240 | 330 | 390 | 420 | 480 | 540 | 570 | 600 | 630 | 660 | NAME | Functional description |
| --- | --- | --- | --- | --- | --- | --- | --- | --- | --- | --- | --- | --- | --- | --- | --- | --- | --- | --- | --- | --- | --- | --- | --- | --- | --- | --- | --- | --- | --- | --- | --- | --- | --- | --- | --- | --- | --- | --- | --- |
|  |  |  |  |  |  |  |  |  |  |  |  |  |  |  |  |  |  |  |  |  |  |  |  |  |  |  |  |  |  |  |  |  |  |  |  |  |  | *col-135* | COLlagen |
|  |  |  |  |  |  |  |  |  |  |  |  |  |  |  |  |  |  |  |  |  |  |  |  |  |  |  |  |  |  |  |  |  |  |  |  |  |  | *pqn-2* | Prion-like-(Q/N-rich)-domain-bearing protein |
|  |  |  |  |  |  |  |  |  |  |  |  |  |  |  |  |  |  |  |  |  |  |  |  |  |  |  |  |  |  |  |  |  |  |  |  |  |  | T13G4.7 |  |
|  |  |  |  |  |  |  |  |  |  |  |  |  |  |  |  |  |  |  |  |  |  |  |  |  |  |  |  |  |  |  |  |  |  |  |  |  |  | R160.4 |  |
|  |  |  |  |  |  |  |  |  |  |  |  |  |  |  |  |  |  |  |  |  |  |  |  |  |  |  |  |  |  |  |  |  |  |  |  |  |  | *anr-13* | Antisense Non-coding RNA |
|  |  |  |  |  |  |  |  |  |  |  |  |  |  |  |  |  |  |  |  |  |  |  |  |  |  |  |  |  |  |  |  |  |  |  |  |  |  | C08A9.6 |  |
|  |  |  |  |  |  |  |  |  |  |  |  |  |  |  |  |  |  |  |  |  |  |  |  |  |  |  |  |  |  |  |  |  |  |  |  |  |  | T27E9.2 |  |
|  |  |  |  |  |  |  |  |  |  |  |  |  |  |  |  |  |  |  |  |  |  |  |  |  |  |  |  |  |  |  |  |  |  |  |  |  |  | Y64H9A.1 |  |
|  |  |  |  |  |  |  |  |  |  |  |  |  |  |  |  |  |  |  |  |  |  |  |  |  |  |  |  |  |  |  |  |  |  |  |  |  |  | C49H3.12 |  |
|  |  |  |  |  |  |  |  |  |  |  |  |  |  |  |  |  |  |  |  |  |  |  |  |  |  |  |  |  |  |  |  |  |  |  |  |  |  | F55G11.6 |  |
|  |  |  |  |  |  |  |  |  |  |  |  |  |  |  |  |  |  |  |  |  |  |  |  |  |  |  |  |  |  |  |  |  |  |  |  |  |  | *far-8* | Fatty Acid/Retinol binding protein |
|  |  |  |  |  |  |  |  |  |  |  |  |  |  |  |  |  |  |  |  |  |  |  |  |  |  |  |  |  |  |  |  |  |  |  |  |  |  | Y39G8B.1 |  |
|  |  |  |  |  |  |  |  |  |  |  |  |  |  |  |  |  |  |  |  |  |  |  |  |  |  |  |  |  |  |  |  |  |  |  |  |  |  | B0250.3 |  |
|  |  |  |  |  |  |  |  |  |  |  |  |  |  |  |  |  |  |  |  |  |  |  |  |  |  |  |  |  |  |  |  |  |  |  |  |  |  | *rps-21* | Ribosomal Protein, Small subunit |
|  |  |  |  |  |  |  |  |  |  |  |  |  |  |  |  |  |  |  |  |  |  |  |  |  |  |  |  |  |  |  |  |  |  |  |  |  |  | *rla-1* | Ribosomal protein, Large subunit, Acidic (P1) |
|  |  |  |  |  |  |  |  |  |  |  |  |  |  |  |  |  |  |  |  |  |  |  |  |  |  |  |  |  |  |  |  |  |  |  |  |  |  | *rpl-34* | Ribosomal Protein, Large subunit |
|  |  |  |  |  |  |  |  |  |  |  |  |  |  |  |  |  |  |  |  |  |  |  |  |  |  |  |  |  |  |  |  |  |  |  |  |  |  | *rps-10* | Ribosomal Protein, Small subunit |
|  |  |  |  |  |  |  |  |  |  |  |  |  |  |  |  |  |  |  |  |  |  |  |  |  |  |  |  |  |  |  |  |  |  |  |  |  |  | *rpl-43* | Ribosomal Protein, Large subunit |
|  |  |  |  |  |  |  |  |  |  |  |  |  |  |  |  |  |  |  |  |  |  |  |  |  |  |  |  |  |  |  |  |  |  |  |  |  |  | *rpl-28* | Ribosomal Protein, Large subunit |
|  |  |  |  |  |  |  |  |  |  |  |  |  |  |  |  |  |  |  |  |  |  |  |  |  |  |  |  |  |  |  |  |  |  |  |  |  |  | *rps-11* | Ribosomal Protein, Small subunit |
|  |  |  |  |  |  |  |  |  |  |  |  |  |  |  |  |  |  |  |  |  |  |  |  |  |  |  |  |  |  |  |  |  |  |  |  |  |  | *rpl-41* | Ribosomal Protein, Large subunit |
|  |  |  |  |  |  |  |  |  |  |  |  |  |  |  |  |  |  |  |  |  |  |  |  |  |  |  |  |  |  |  |  |  |  |  |  |  |  | *rpl-16* | Ribosomal Protein, Large subunit |
|  |  |  |  |  |  |  |  |  |  |  |  |  |  |  |  |  |  |  |  |  |  |  |  |  |  |  |  |  |  |  |  |  |  |  |  |  |  | M01F1.3 |  |
|  |  |  |  |  |  |  |  |  |  |  |  |  |  |  |  |  |  |  |  |  |  |  |  |  |  |  |  |  |  |  |  |  |  |  |  |  |  | *rpl-35* | Ribosomal Protein, Large subunit |
|  |  |  |  |  |  |  |  |  |  |  |  |  |  |  |  |  |  |  |  |  |  |  |  |  |  |  |  |  |  |  |  |  |  |  |  |  |  | *rpl-20* | Ribosomal Protein, Large subunit |
|  |  |  |  |  |  |  |  |  |  |  |  |  |  |  |  |  |  |  |  |  |  |  |  |  |  |  |  |  |  |  |  |  |  |  |  |  |  | *rps-19* | Ribosomal Protein, Small subunit |
|  |  |  |  |  |  |  |  |  |  |  |  |  |  |  |  |  |  |  |  |  |  |  |  |  |  |  |  |  |  |  |  |  |  |  |  |  |  | W01D2.1 |  |
|  |  |  |  |  |  |  |  |  |  |  |  |  |  |  |  |  |  |  |  |  |  |  |  |  |  |  |  |  |  |  |  |  |  |  |  |  |  | *rpl-38* | Ribosomal Protein, Large subunit |
|  |  |  |  |  |  |  |  |  |  |  |  |  |  |  |  |  |  |  |  |  |  |  |  |  |  |  |  |  |  |  |  |  |  |  |  |  |  | *rpl-15* | Ribosomal Protein, Large subunit |
|  |  |  |  |  |  |  |  |  |  |  |  |  |  |  |  |  |  |  |  |  |  |  |  |  |  |  |  |  |  |  |  |  |  |  |  |  |  | *rpl-24.1* | Ribosomal Protein, Large subunit |
|  |  |  |  |  |  |  |  |  |  |  |  |  |  |  |  |  |  |  |  |  |  |  |  |  |  |  |  |  |  |  |  |  |  |  |  |  |  | *rpl-19* | Ribosomal Protein, Large subunit |
|  |  |  |  |  |  |  |  |  |  |  |  |  |  |  |  |  |  |  |  |  |  |  |  |  |  |  |  |  |  |  |  |  |  |  |  |  |  | *rpl-14* | Ribosomal Protein, Large subunit |
|  |  |  |  |  |  |  |  |  |  |  |  |  |  |  |  |  |  |  |  |  |  |  |  |  |  |  |  |  |  |  |  |  |  |  |  |  |  | *rpl-26* | Ribosomal Protein, Large subunit |
|  |  |  |  |  |  |  |  |  |  |  |  |  |  |  |  |  |  |  |  |  |  |  |  |  |  |  |  |  |  |  |  |  |  |  |  |  |  | *rpl-30* | Ribosomal Protein, Large subunit |
|  |  |  |  |  |  |  |  |  |  |  |  |  |  |  |  |  |  |  |  |  |  |  |  |  |  |  |  |  |  |  |  |  |  |  |  |  |  | *rps-4* | Ribosomal Protein, Small subunit |
|  |  |  |  |  |  |  |  |  |  |  |  |  |  |  |  |  |  |  |  |  |  |  |  |  |  |  |  |  |  |  |  |  |  |  |  |  |  | *rps-26* | Ribosomal Protein, Small subunit |
|  |  |  |  |  |  |  |  |  |  |  |  |  |  |  |  |  |  |  |  |  |  |  |  |  |  |  |  |  |  |  |  |  |  |  |  |  |  | *rpl-36* | Ribosomal Protein, Large subunit |
|  |  |  |  |  |  |  |  |  |  |  |  |  |  |  |  |  |  |  |  |  |  |  |  |  |  |  |  |  |  |  |  |  |  |  |  |  |  | *rps-28* | Ribosomal Protein, Small subunit |
|  |  |  |  |  |  |  |  |  |  |  |  |  |  |  |  |  |  |  |  |  |  |  |  |  |  |  |  |  |  |  |  |  |  |  |  |  |  | *rps-27* | Ribosomal Protein, Small subunit |
|  |  |  |  |  |  |  |  |  |  |  |  |  |  |  |  |  |  |  |  |  |  |  |  |  |  |  |  |  |  |  |  |  |  |  |  |  |  | *rla-2* | Ribosomal protein, Large subunit, Acidic (P1) |
|  |  |  |  |  |  |  |  |  |  |  |  |  |  |  |  |  |  |  |  |  |  |  |  |  |  |  |  |  |  |  |  |  |  |  |  |  |  | F54D7.7 |  |
|  |  |  |  |  |  |  |  |  |  |  |  |  |  |  |  |  |  |  |  |  |  |  |  |  |  |  |  |  |  |  |  |  |  |  |  |  |  | *rpl-18* | Ribosomal Protein, Large subunit |
|  |  |  |  |  |  |  |  |  |  |  |  |  |  |  |  |  |  |  |  |  |  |  |  |  |  |  |  |  |  |  |  |  |  |  |  |  |  | *rpl-9* | Ribosomal Protein, Large subunit |
|  |  |  |  |  |  |  |  |  |  |  |  |  |  |  |  |  |  |  |  |  |  |  |  |  |  |  |  |  |  |  |  |  |  |  |  |  |  | *rpl-2* | Ribosomal Protein, Large subunit |
|  |  |  |  |  |  |  |  |  |  |  |  |  |  |  |  |  |  |  |  |  |  |  |  |  |  |  |  |  |  |  |  |  |  |  |  |  |  | *ubq-2* | UBiQuitin |
|  |  |  |  |  |  |  |  |  |  |  |  |  |  |  |  |  |  |  |  |  |  |  |  |  |  |  |  |  |  |  |  |  |  |  |  |  |  | *rps-25* | Ribosomal Protein, Small subunit |
|  |  |  |  |  |  |  |  |  |  |  |  |  |  |  |  |  |  |  |  |  |  |  |  |  |  |  |  |  |  |  |  |  |  |  |  |  |  | *rps-29* | Ribosomal Protein, Small subunit |
|  |  |  |  |  |  |  |  |  |  |  |  |  |  |  |  |  |  |  |  |  |  |  |  |  |  |  |  |  |  |  |  |  |  |  |  |  |  | *rpl-29* | Ribosomal Protein, Large subunit |
|  |  |  |  |  |  |  |  |  |  |  |  |  |  |  |  |  |  |  |  |  |  |  |  |  |  |  |  |  |  |  |  |  |  |  |  |  |  | C08F8.9 |  |
|  |  |  |  |  |  |  |  |  |  |  |  |  |  |  |  |  |  |  |  |  |  |  |  |  |  |  |  |  |  |  |  |  |  |  |  |  |  | Y82E9BR.3 |  |
|  |  |  |  |  |  |  |  |  |  |  |  |  |  |  |  |  |  |  |  |  |  |  |  |  |  |  |  |  |  |  |  |  |  |  |  |  |  | *mrpl-36* | Mitochondrial Ribosomal Protein, Large |
|  |  |  |  |  |  |  |  |  |  |  |  |  |  |  |  |  |  |  |  |  |  |  |  |  |  |  |  |  |  |  |  |  |  |  |  |  |  | Y54G2A.18 |  |
|  |  |  |  |  |  |  |  |  |  |  |  |  |  |  |  |  |  |  |  |  |  |  |  |  |  |  |  |  |  |  |  |  |  |  |  |  |  | *swt-7* | SWEET sugar transporter family |
|  |  |  |  |  |  |  |  |  |  |  |  |  |  |  |  |  |  |  |  |  |  |  |  |  |  |  |  |  |  |  |  |  |  |  |  |  |  | *pbs-1* | Proteasome Beta Subunit |
|  |  |  |  |  |  |  |  |  |  |  |  |  |  |  |  |  |  |  |  |  |  |  |  |  |  |  |  |  |  |  |  |  |  |  |  |  |  | W08G11.6 |  |
|  |  |  |  |  |  |  |  |  |  |  |  |  |  |  |  |  |  |  |  |  |  |  |  |  |  |  |  |  |  |  |  |  |  |  |  |  |  | *nstp-4* | Nucleotide Sugar TransPorter family |
|  |  |  |  |  |  |  |  |  |  |  |  |  |  |  |  |  |  |  |  |  |  |  |  |  |  |  |  |  |  |  |  |  |  |  |  |  |  | F11A10.6 |  |
|  |  |  |  |  |  |  |  |  |  |  |  |  |  |  |  |  |  |  |  |  |  |  |  |  |  |  |  |  |  |  |  |  |  |  |  |  |  | *tag-335* | Temporarily Assigned Gene name |
|  |  |  |  |  |  |  |  |  |  |  |  |  |  |  |  |  |  |  |  |  |  |  |  |  |  |  |  |  |  |  |  |  |  |  |  |  |  | *ndx-3* | NuDiX family |
|  |  |  |  |  |  |  |  |  |  |  |  |  |  |  |  |  |  |  |  |  |  |  |  |  |  |  |  |  |  |  |  |  |  |  |  |  |  | F35G2.2 |  |
|  |  |  |  |  |  |  |  |  |  |  |  |  |  |  |  |  |  |  |  |  |  |  |  |  |  |  |  |  |  |  |  |  |  |  |  |  |  | R10E4.9 |  |
|  |  |  |  |  |  |  |  |  |  |  |  |  |  |  |  |  |  |  |  |  |  |  |  |  |  |  |  |  |  |  |  |  |  |  |  |  |  | F20G2.2 |  |
|  |  |  |  |  |  |  |  |  |  |  |  |  |  |  |  |  |  |  |  |  |  |  |  |  |  |  |  |  |  |  |  |  |  |  |  |  |  | *fbxa-77* | F-box A protein |
|  |  |  |  |  |  |  |  |  |  |  |  |  |  |  |  |  |  |  |  |  |  |  |  |  |  |  |  |  |  |  |  |  |  |  |  |  |  | *srxa-18* | Serpentine Receptor, class XA |
|  |  |  |  |  |  |  |  |  |  |  |  |  |  |  |  |  |  |  |  |  |  |  |  |  |  |  |  |  |  |  |  |  |  |  |  |  |  | *lec-12* | gaLECtin |
|  |  |  |  |  |  |  |  |  |  |  |  |  |  |  |  |  |  |  |  |  |  |  |  |  |  |  |  |  |  |  |  |  |  |  |  |  |  | Y71H2AR.1 |  |
|  |  |  |  |  |  |  |  |  |  |  |  |  |  |  |  |  |  |  |  |  |  |  |  |  |  |  |  |  |  |  |  |  |  |  |  |  |  | *fkb-1* | FK506-Binding protein family |
|  |  |  |  |  |  |  |  |  |  |  |  |  |  |  |  |  |  |  |  |  |  |  |  |  |  |  |  |  |  |  |  |  |  |  |  |  |  | Y38F2AR.9 |  |
|  |  |  |  |  |  |  |  |  |  |  |  |  |  |  |  |  |  |  |  |  |  |  |  |  |  |  |  |  |  |  |  |  |  |  |  |  |  | *trap-3* | TRanslocon-Associated Protein |
|  |  |  |  |  |  |  |  |  |  |  |  |  |  |  |  |  |  |  |  |  |  |  |  |  |  |  |  |  |  |  |  |  |  |  |  |  |  | *emo-1* | EndoMitotic Oocytes |
|  |  |  |  |  |  |  |  |  |  |  |  |  |  |  |  |  |  |  |  |  |  |  |  |  |  |  |  |  |  |  |  |  |  |  |  |  |  | *trap-2* | TRanslocon-Associated Protein |
|  |  |  |  |  |  |  |  |  |  |  |  |  |  |  |  |  |  |  |  |  |  |  |  |  |  |  |  |  |  |  |  |  |  |  |  |  |  | *lbp-9* | Lipid Binding Protein |
|  |  |  |  |  |  |  |  |  |  |  |  |  |  |  |  |  |  |  |  |  |  |  |  |  |  |  |  |  |  |  |  |  |  |  |  |  |  | F59F4.2 |  |
|  |  |  |  |  |  |  |  |  |  |  |  |  |  |  |  |  |  |  |  |  |  |  |  |  |  |  |  |  |  |  |  |  |  |  |  |  |  | H01M10.1 |  |
|  |  |  |  |  |  |  |  |  |  |  |  |  |  |  |  |  |  |  |  |  |  |  |  |  |  |  |  |  |  |  |  |  |  |  |  |  |  | M163.1 |  |
|  |  |  |  |  |  |  |  |  |  |  |  |  |  |  |  |  |  |  |  |  |  |  |  |  |  |  |  |  |  |  |  |  |  |  |  |  |  | ZK688.7 |  |
|  |  |  |  |  |  |  |  |  |  |  |  |  |  |  |  |  |  |  |  |  |  |  |  |  |  |  |  |  |  |  |  |  |  |  |  |  |  | *nhr-122* | Nuclear Hormone Receptor family |
|  |  |  |  |  |  |  |  |  |  |  |  |  |  |  |  |  |  |  |  |  |  |  |  |  |  |  |  |  |  |  |  |  |  |  |  |  |  | C50H2.13 |  |
|  |  |  |  |  |  |  |  |  |  |  |  |  |  |  |  |  |  |  |  |  |  |  |  |  |  |  |  |  |  |  |  |  |  |  |  |  |  | C30G4.4 |  |
|  |  |  |  |  |  |  |  |  |  |  |  |  |  |  |  |  |  |  |  |  |  |  |  |  |  |  |  |  |  |  |  |  |  |  |  |  |  | VF15C11L.2 |  |
|  |  |  |  |  |  |  |  |  |  |  |  |  |  |  |  |  |  |  |  |  |  |  |  |  |  |  |  |  |  |  |  |  |  |  |  |  |  | F55B12.10 |  |
|  |  |  |  |  |  |  |  |  |  |  |  |  |  |  |  |  |  |  |  |  |  |  |  |  |  |  |  |  |  |  |  |  |  |  |  |  |  | F53B2.5 |  |
|  |  |  |  |  |  |  |  |  |  |  |  |  |  |  |  |  |  |  |  |  |  |  |  |  |  |  |  |  |  |  |  |  |  |  |  |  |  | *clec-136* | C-type LECtin |
|  |  |  |  |  |  |  |  |  |  |  |  |  |  |  |  |  |  |  |  |  |  |  |  |  |  |  |  |  |  |  |  |  |  |  |  |  |  | *grl-22* | GRound-Like (grd related) |
|  |  |  |  |  |  |  |  |  |  |  |  |  |  |  |  |  |  |  |  |  |  |  |  |  |  |  |  |  |  |  |  |  |  |  |  |  |  | K08E5.5 |  |
|  |  |  |  |  |  |  |  |  |  |  |  |  |  |  |  |  |  |  |  |  |  |  |  |  |  |  |  |  |  |  |  |  |  |  |  |  |  | T23B3.6 |  |
|  |  |  |  |  |  |  |  |  |  |  |  |  |  |  |  |  |  |  |  |  |  |  |  |  |  |  |  |  |  |  |  |  |  |  |  |  |  | H16D19.7 |  |
|  |  |  |  |  |  |  |  |  |  |  |  |  |  |  |  |  |  |  |  |  |  |  |  |  |  |  |  |  |  |  |  |  |  |  |  |  |  | K01A6.17 |  |
|  |  |  |  |  |  |  |  |  |  |  |  |  |  |  |  |  |  |  |  |  |  |  |  |  |  |  |  |  |  |  |  |  |  |  |  |  |  | C24A11.13 |  |
|  |  |  |  |  |  |  |  |  |  |  |  |  |  |  |  |  |  |  |  |  |  |  |  |  |  |  |  |  |  |  |  |  |  |  |  |  |  | C06E4.15 |  |
|  |  |  |  |  |  |  |  |  |  |  |  |  |  |  |  |  |  |  |  |  |  |  |  |  |  |  |  |  |  |  |  |  |  |  |  |  |  | *tth-1* | Tetra THymosin (four thymosin repeat protein) |
|  |  |  |  |  |  |  |  |  |  |  |  |  |  |  |  |  |  |  |  |  |  |  |  |  |  |  |  |  |  |  |  |  |  |  |  |  |  | T28F4.5 |  |
|  |  |  |  |  |  |  |  |  |  |  |  |  |  |  |  |  |  |  |  |  |  |  |  |  |  |  |  |  |  |  |  |  |  |  |  |  |  | Y57E12AR.1 |  |
|  |  |  |  |  |  |  |  |  |  |  |  |  |  |  |  |  |  |  |  |  |  |  |  |  |  |  |  |  |  |  |  |  |  |  |  |  |  | F13H8.9 |  |
|  |  |  |  |  |  |  |  |  |  |  |  |  |  |  |  |  |  |  |  |  |  |  |  |  |  |  |  |  |  |  |  |  |  |  |  |  |  | M01A8.1 |  |
|  |  |  |  |  |  |  |  |  |  |  |  |  |  |  |  |  |  |  |  |  |  |  |  |  |  |  |  |  |  |  |  |  |  |  |  |  |  | *flp-15* | FMRF-Like Peptide |
|  |  |  |  |  |  |  |  |  |  |  |  |  |  |  |  |  |  |  |  |  |  |  |  |  |  |  |  |  |  |  |  |  |  |  |  |  |  | F46B3.8 |  |
|  |  |  |  |  |  |  |  |  |  |  |  |  |  |  |  |  |  |  |  |  |  |  |  |  |  |  |  |  |  |  |  |  |  |  |  |  |  | *mom-1* | More Of MS |
|  |  |  |  |  |  |  |  |  |  |  |  |  |  |  |  |  |  |  |  |  |  |  |  |  |  |  |  |  |  |  |  |  |  |  |  |  |  | BE10.6 |  |
|  |  |  |  |  |  |  |  |  |  |  |  |  |  |  |  |  |  |  |  |  |  |  |  |  |  |  |  |  |  |  |  |  |  |  |  |  |  | W03G11.7 |  |
|  |  |  |  |  |  |  |  |  |  |  |  |  |  |  |  |  |  |  |  |  |  |  |  |  |  |  |  |  |  |  |  |  |  |  |  |  |  | *clec-266* | C-type LECtin |
|  |  |  |  |  |  |  |  |  |  |  |  |  |  |  |  |  |  |  |  |  |  |  |  |  |  |  |  |  |  |  |  |  |  |  |  |  |  | ZK822.2 |  |
|  |  |  |  |  |  |  |  |  |  |  |  |  |  |  |  |  |  |  |  |  |  |  |  |  |  |  |  |  |  |  |  |  |  |  |  |  |  | R11G1.2 |  |
|  |  |  |  |  |  |  |  |  |  |  |  |  |  |  |  |  |  |  |  |  |  |  |  |  |  |  |  |  |  |  |  |  |  |  |  |  |  | K12H6.7 |  |
|  |  |  |  |  |  |  |  |  |  |  |  |  |  |  |  |  |  |  |  |  |  |  |  |  |  |  |  |  |  |  |  |  |  |  |  |  |  | *nrf-6* | Nose Resistant to Fluoxetine |
|  |  |  |  |  |  |  |  |  |  |  |  |  |  |  |  |  |  |  |  |  |  |  |  |  |  |  |  |  |  |  |  |  |  |  |  |  |  | *gpx-5* | Glutathione PeroXidase |
|  |  |  |  |  |  |  |  |  |  |  |  |  |  |  |  |  |  |  |  |  |  |  |  |  |  |  |  |  |  |  |  |  |  |  |  |  |  | C01G10.15 |  |
|  |  |  |  |  |  |  |  |  |  |  |  |  |  |  |  |  |  |  |  |  |  |  |  |  |  |  |  |  |  |  |  |  |  |  |  |  |  | F58D2.4 |  |
|  |  |  |  |  |  |  |  |  |  |  |  |  |  |  |  |  |  |  |  |  |  |  |  |  |  |  |  |  |  |  |  |  |  |  |  |  |  | *anr-31* | Antisense Non-coding RNA |

### Phenotypes enriched

|  |  |  |  |
| --- | --- | --- | --- |
| **Group name** | **Number in cluster** | **Enrichment** | **FDR corrected p** |
| pleiotropic defects severe early emb (RNAi) | 27 | 31.33 | 1.57e-28 |
| other abnormality early emb (RNAi) | 27 | 24.74 | 1.19e-25 |
| early embryonic lethal (RNAi) | 31 | 4.57 | 2.18e-09 |
| protein aggregation variant (RNAi) | 19 | 5.45 | 5.24e-06 |
| protein interaction variant (RNAi) | 19 | 5.41 | 5.59e-06 |
| protein protein interaction variant (RNAi) | 19 | 5.41 | 5.59e-06 |
| pathogen resistance increased (RNAi) | 7 | 22.38 | 6.11e-05 |
| maternal sterile (RNAi) | 26 | 3.38 | 6.82e-05 |
| hermaphrodite sterile (RNAi) | 26 | 3.38 | 6.90e-05 |
| hermaphrodite fertility reduced (RNAi) | 26 | 3.38 | 6.90e-05 |
| larval arrest (RNAi) | 33 | 2.67 | 1.68e-04 |
| developmental growth variant (RNAi) | 33 | 2.67 | 1.70e-04 |
| larval growth variant (RNAi) | 33 | 2.67 | 1.72e-04 |
| larval development variant (RNAi) | 34 | 2.55 | 2.88e-04 |
| postembryonic development variant (RNAi) | 34 | 2.42 | 9.10e-04 |
| transgene subcellular localization variant (RNAi) | 17 | 4.12 | 1.24e-03 |
| organism pathogen response variant (RNAi) | 7 | 8.68 | 1.94e-02 |
| growth variant (RNAi) | 40 | 1.83 | 4.29e-02 |

### Anatomy terms enriched

none found

### GO terms enriched

|  |  |  |
| --- | --- | --- |
| **GO term** | **Number of genes** | **FDR-corrected p-value** |
| structural constituent of ribosome | 27 | 1.6e-32 |
| ribosome | 25 | 1.2e-30 |
| translation | 24 | 2.9e-22 |
| macromolecular complex | 32 | 2.4e-11 |
| non-membrane-bounded organelle | 29 | 3.8e-11 |
| cytoplasm | 38 | 5.3e-10 |
| cellular biosynthetic process | 32 | 4.4e-09 |
| macromolecule biosynthetic process | 29 | 6.9e-09 |
| protein metabolic process | 30 | 2.0e-08 |
| growth | 17 | 4.5e-06 |
| embryo development ending in birth or egg hatching | 37 | 1.8e-05 |
| intracellular organelle | 39 | 3.1e-05 |
| nematode larval development | 30 | 1.2e-04 |
| cellular macromolecule metabolic process | 31 | 1.2e-04 |
| post-embryonic development | 30 | 1.4e-04 |
| anatomical structure development | 40 | 6.1e-04 |
| intracellular | 40 | 1.6e-03 |
| molting cycle, collagen and cuticulin-based cuticle | 10 | 2.3e-03 |
| apoptotic process | 12 | 4.8e-03 |
| cell death | 12 | 1.4e-02 |
| organic substance metabolic process | 35 | 1.4e-02 |
| cell | 41 | 1.9e-02 |
| translational elongation | 3 | 3.9e-02 |

### Expression clusters enriched

|  |  |  |  |
| --- | --- | --- | --- |
| **Group name** | **Number in cluster** | **Enrichment** | **FDR corrected p** |
| C-lineage related expression profile. WBPaper00025032:cluster\_10 | 22 | 35.97 | 1.71e-25 |
| Differentially expressed genes during worm lifespan. Medoid 7 Fig.4. | 27 | 11.70 | 2.09e-18 |
| Genes in the top 10% of expression level across the triplicate L3 samples. To generate the top10 and bottom10 gene sets, authors ranked all genes by mean expression array signal intensity across the three replicates, then took the top and bottom deciles (1,841 genes each) to represent genes with high and low expression. | 51 | 3.99 | 1.10e-16 |
| Early embryonic development gene expression profile. [cgc5767]:cluster\_2 | 22 | 7.47 | 1.20e-10 |
| Maternal-embryonic class (ME): genes that are in the intersection of the maternal and embryonic classes. | 50 | 2.72 | 1.26e-09 |
| Embryonic (E) subclasses are based on the earliest significant increase(abbreviated pi for primary increase). [cgc5767]:expression\_class\_E\_pi(66\_min) | 17 | 8.74 | 6.58e-09 |
| The cluster contains genes that are significantly enriched in L1 muscle. | 33 | 3.29 | 2.63e-07 |
| Genes downregulated in daf-2(-) compared to daf-16(-); daf-2(-) | 14 | 7.07 | 5.68e-06 |
| C-lineage related expression profile. WBPaper00025032:cluster\_25 | 7 | 22.38 | 1.19e-05 |
| Embryonic class (E): genes that significantly increase in abundance at some point during embryogenesis. | 51 | 2.06 | 1.29e-05 |
| Genes up or down regulated by 10e-09M of cholesterol . The normalized values used were G/R ratio > 2.6 for up-regulation and G/R ratio < 0.38 for down-regulation, which corresponds to 1.39 and -1.39 log(base2) G/R ratio, respectively. | 33 | 2.65 | 3.94e-05 |
| Genes up or down regulated by 10e-05M of estrogen. The normalized values used were G/R ratio > 2.6 for up-regulation and G/R ratio < 0.38 for down-regulation, which corresponds to 1.39 and -1.39 log(base2) G/R ratio, respectively. | 27 | 2.94 | 1.01e-04 |
| Early embryonic development gene expression profile. [cgc5767]:cluster\_16 | 5 | 24.81 | 5.77e-04 |
| Caenorhabditis elegans Genes with expression levels changed significantly after treatment of Bacillus thurigiensis DB27. | 57 | 1.71 | 6.20e-04 |
| Genes that are up or down regulated by more than 2.1 fold with the t-test p-value less than 0.01 are included in this cluster. | 24 | 2.81 | 1.06e-03 |
| Caenorhabditis elegans Genes with expression levels changed significantly after treatment of Serratia marcescens. | 46 | 1.87 | 1.28e-03 |
| Genome-wide analysis of developmental and sex-regulated gene expression profile. cgc4489\_group\_3 | 18 | 3.33 | 2.05e-03 |
| Genes up or down regulated by 10e-05M of progesterone. The normalized values used were G/R ratio > 2.6 for up-regulation and G/R ratio < 0.38 for down-regulation, which corresponds to 1.39 and -1.39 log(base2) G/R ratio, respectively. | 35 | 2.10 | 2.53e-03 |
| Genes predicted to be downregulated more than 2.0 fold in (AFD+AWB) datasets as compared to unsorted whole embryonic cells dataset. | 14 | 3.83 | 4.86e-03 |
| Gene up regulated by chemically-defined medium (CeMM). | 4 | 23.02 | 7.03e-03 |
| Genes enriched in muscle. | 12 | 4.17 | 8.37e-03 |
| C-lineage related expression profile. WBPaper00025032:cluster\_120 | 3 | 39.24 | 1.33e-02 |
| C-lineage related expression profile. WBPaper00025032:cluster\_107 | 3 | 33.20 | 2.15e-02 |
| Genes with decreased expression levels (P < 0.05) under ifg-1 inhibition. | 4 | 15.99 | 2.60e-02 |

### Motifs enriched

|  |  |  |  |  |  |
| --- | --- | --- | --- | --- | --- |
| **Motif** | **Logo** | **Possible orthologs** | **Number of motifs in cluster** | **Enrichment** | **FDR corrected p** |
| pTH10810 |  | lsy-2 (-0.61) syd-9 (-0.6) lsl-1 | 33 | 2.88 | 5.9e-06 |
| Mv109 |  | pax-2 | 34 | 2.63 | 2.6e-05 |
| MA0049.1 |  | hbl-1 (-0.58) | 54 | 1.89 | 5.0e-05 |
| ONECUT2\_1 |  | ceh-48 (-0.72) dsc-1 | 59 | 1.80 | 5.1e-05 |
| pTH6106 |  | nhr-182 | 55 | 1.80 | 1.7e-04 |
| MA0146.2 |  | F58G1.2 | 37 | 2.23 | 2.4e-04 |
| rn\_SOLEXA\_5\_FBgn0259172 |  | lin-29 | 59 | 1.69 | 3.5e-04 |
| pTH2933 |  | F58G1.2 | 33 | 2.34 | 3.9e-04 |
| CG2052\_SANGER\_2.5\_FBgn0039905 |  | fkh-7 lin-29 | 66 | 1.59 | 4.2e-04 |
| Eip93F\_SANGER\_10\_FBgn0013948 |  | mbr-1 (-0.56) bed-3 nhr-177 | 70 | 1.54 | 4.5e-04 |
| pTH8649 |  | mbr-1 (-0.56) | 63 | 1.62 | 4.5e-04 |
| pTH9220 |  | mbr-1 (-0.56) | 63 | 1.61 | 6.1e-04 |
| pTH9097 |  | Y116A8C.22 | 59 | 1.65 | 7.4e-04 |
| pTH8863 |  | hmg-12 | 58 | 1.62 | 1.5e-03 |
| Mv90 |  | mef-2 | 51 | 1.72 | 1.7e-03 |
| pTH10797 |  | K11D2.4 lin-29 | 55 | 1.65 | 1.8e-03 |
| Foxg1\_2 |  | lin-31 | 33 | 2.15 | 1.8e-03 |
| pTH9380 |  | mel-28 | 46 | 1.80 | 1.8e-03 |
| MA0547.1 |  | skn-1 (-0.63) | 79 | 1.39 | 1.9e-03 |
| Foxl1\_2809 |  | daf-16 (-0.56) let-381 fkh-8 lin-31 fkh-7 pha-4 | 75 | 1.43 | 1.9e-03 |
| CG4328\_Cell\_FBgn0036274 |  | lim-6 alr-1 | 26 | 2.43 | 2.3e-03 |
| pTH5260 |  | aha-1 (-0.58) lin-22 | 44 | 1.82 | 2.4e-03 |
| tgo\_tai\_SANGER\_5\_FBgn0015014 |  | aha-1 (-0.58) hlh-30 | 54 | 1.65 | 2.4e-03 |
| pTH8982 |  | ceh-48 (-0.72) | 39 | 1.90 | 3.1e-03 |
| pTH6003 |  | nhr-182 nhr-134 | 59 | 1.56 | 3.6e-03 |
| V$SP1\_01 |  | klf-1 klf-2 | 50 | 1.68 | 3.6e-03 |
| V$NKX61\_01 |  | ceh-31 cog-1 | 34 | 2.02 | 3.9e-03 |
| pTH9082 |  | mab-23 | 72 | 1.42 | 4.4e-03 |
| Hr78\_SANGER\_5\_FBgn0015239 |  | nhr-19 nhr-10 | 37 | 1.91 | 4.8e-03 |
| pTH9335 |  | mel-28 | 47 | 1.70 | 5.0e-03 |
| Klf7\_0974 |  | klf-1 | 46 | 1.71 | 5.4e-03 |
| MGA\_2 |  | tbx-42 | 36 | 1.91 | 5.9e-03 |
| Blimp-1\_SANGER\_5\_FBgn0035625 |  | blmp-1 | 80 | 1.34 | 6.3e-03 |
| Foxa2\_2830 |  | daf-16 (-0.56) fkh-10 let-381 lin-31 fkh-7 | 65 | 1.46 | 6.6e-03 |
| POU4F1\_1 |  | unc-86 | 70 | 1.41 | 8.2e-03 |
| eve\_FlyReg\_FBgn0000606 |  | ceh-53 hmbx-1 Y116A8C.22 | 70 | 1.41 | 8.3e-03 |
| E2F4\_1 |  | nfi-1 (-0.61) F49E12.6 | 57 | 1.53 | 8.8e-03 |
| Fkh2 |  | fkh-10 let-381 lin-31 | 68 | 1.42 | 8.9e-03 |
| pTH8985 |  | athp-1 | 37 | 1.84 | 9.4e-03 |
| pTH10647 |  | nhr-232 | 21 | 2.48 | 9.5e-03 |
| HOXC10\_3 |  | php-3 ceh-13 lin-39 D1005.3 | 64 | 1.45 | 9.7e-03 |
| MA0544.1 |  | gei-11 | 45 | 1.68 | 9.9e-03 |
| KLF4\_f2 |  | klf-1 klf-2 | 46 | 1.66 | 9.9e-03 |
| pTH9173 |  | efl-2 | 27 | 2.13 | 1.0e-02 |
| Ara\_SOLEXA\_FBgn0015904 |  | daf-16 (-0.56) irx-1 | 67 | 1.42 | 1.0e-02 |
| pTH9254 |  | mel-28 | 48 | 1.62 | 1.1e-02 |
| ARI3A\_do |  | cfi-1 | 84 | 1.29 | 1.1e-02 |
| MA0459.1 |  | nhr-239 | 68 | 1.41 | 1.2e-02 |
| HES1\_f1 |  | lin-22 | 45 | 1.66 | 1.2e-02 |
| MSX2\_f1 |  | ceh-1 | 48 | 1.61 | 1.3e-02 |
| pTH9085 |  | nhr-42 | 36 | 1.82 | 1.3e-02 |
| Eip75B\_SANGER\_5\_FBgn0000568 |  | nhr-118 nhr-213 | 46 | 1.64 | 1.3e-02 |
| MA0142.1 |  | sox-4 (-0.59) ceh-6 | 48 | 1.60 | 1.4e-02 |
| ERG\_4 |  | lin-1 | 41 | 1.70 | 1.6e-02 |
| pTH6486 |  | nhr-145 (-0.57) | 61 | 1.45 | 1.6e-02 |
| V$TAXCREB\_01 |  | crh-1 | 21 | 2.36 | 1.6e-02 |
| Hoxa11\_2218 |  | php-3 | 51 | 1.55 | 1.8e-02 |
| HOXC10\_2 |  | php-3 lin-39 | 65 | 1.41 | 1.8e-02 |
| Zfp161\_2858 |  | pzf-1 | 33 | 1.86 | 1.8e-02 |
| ss\_tgo\_SANGER\_10\_FBgn0015014 |  | aha-1 (-0.58) | 38 | 1.74 | 1.9e-02 |
| V$BRN2\_01 |  | ceh-18 | 79 | 1.30 | 1.9e-02 |
| V$ARNT\_02 |  | aha-1 (-0.58) mxl-2 mxl-1 ref-1 lin-22 hlh-30 C27D6.4 | 11 | 3.59 | 2.1e-02 |
| ARX\_1 |  | alr-1 | 61 | 1.43 | 2.1e-02 |
| pTH8998 |  | mab-3 | 55 | 1.49 | 2.2e-02 |
| pTH5916 |  | efl-2 | 38 | 1.72 | 2.3e-02 |
| GSE15244\_FoxA1 |  | let-381 lin-31 | 76 | 1.31 | 2.5e-02 |
| HIF1A\_si |  | hif-1 (-0.59) | 43 | 1.62 | 2.6e-02 |
| MA0536.1 |  | elt-1 | 32 | 1.83 | 2.7e-02 |
| SMAD3\_1 |  | daf-8 | 19 | 2.37 | 2.8e-02 |
| pTH1294 |  | mel-28 | 41 | 1.64 | 2.8e-02 |
| MAX\_1 |  | mxl-1 | 22 | 2.18 | 2.8e-02 |
| MA0071.1 |  | nhr-213 | 49 | 1.53 | 3.1e-02 |
| pTH9245 |  | ceh-18 | 13 | 2.94 | 3.4e-02 |
| pTH2280 |  | mnm-2 | 22 | 2.13 | 3.5e-02 |
| HMGA1\_f1 |  | let-381 Y116A8C.22 | 71 | 1.32 | 4.0e-02 |
| Dll\_Cell\_FBgn0000157 |  | ceh-43 | 60 | 1.40 | 4.1e-02 |
| pTH10823 |  | B0310.2 | 16 | 2.49 | 4.2e-02 |
| pTH3046 |  | Y116A8C.22 | 50 | 1.49 | 4.4e-02 |
| BARHL2\_4 |  | ceh-31 | 10 | 3.44 | 4.4e-02 |
| ZNF75A\_1 |  | ztf-3 (-0.55) | 20 | 2.16 | 4.9e-02 |
| Hr51\_SANGER\_5\_FBgn0034012 |  | nhr-100 | 41 | 1.59 | 5.0e-02 |

### Correlated (and anti-correlated) transcription factors

|  |  |
| --- | --- |
| **Transcription factor** | **Correlation** |
| nhr-122 | 0.75 |
| grh-1 | 0.73 |
| atf-8 | 0.65 |
| hlh-12 | 0.65 |
| nhr-168 | 0.62 |
| nhr-204 | 0.60 |
| nhr-104 | 0.59 |
| pros-1 | 0.56 |
| Y56A3A.18 | 0.55 |
| nhr-146 | 0.54 |
| nhr-87 | 0.54 |
| nhr-23 | 0.52 |
| C02F12.5 | 0.52 |
| ceh-7 | 0.51 |
| ceh-82 | 0.51 |
| zip-6 | 0.51 |
| ztf-27 | 0.51 |
| nhr-140 | 0.48 |
| nhr-112 | 0.48 |
| let-381 | 0.48 |
| nhr-90 | 0.47 |
| nhr-55 | 0.47 |
| mxl-2 | 0.47 |
| nhr-70 | 0.46 |
| blmp-1 | 0.46 |
| spr-4 | -0.72 |
| lim-4 | -0.72 |
| Y17G7B.22 | -0.72 |
| nhr-86 | -0.72 |
| let-526 | -0.72 |
| ceh-48 | -0.72 |
| ceh-36 | -0.73 |
| tag-146 | -0.74 |
| aptf-4 | -0.74 |
| F56D1.1 | -0.74 |
| ceh-38 | -0.75 |
| ZK546.5 | -0.75 |
| miz-1 | -0.76 |
| ceh-44 | -0.76 |
| ceh-93 | -0.76 |
| mgl-2 | -0.77 |
| ceh-89 | -0.77 |
| wrm-1 | -0.77 |
| sem-4 | -0.77 |
| C34F6.9 | -0.78 |
| dhhc-14 | -0.78 |
| athp-2 | -0.79 |
| nhr-47 | -0.82 |
| C17E4.6 | -0.87 |
| C33H5.17 | -0.88 |

### ChIP peaks enriched

|  |  |  |  |  |
| --- | --- | --- | --- | --- |
| **Gene** | **Experiment** | **Number of upstream peaks** | **Enrichment** | **FDR corrected p** |
| lin-15 | LIN-15B\_Larvae-L4-stage | 31 | 9.35 | 3.4e-19 |
| nfya-1 | NFYA-1\_Young-adult | 36 | 7.24 | 4.4e-19 |
| lin-13 | LIN-13\_Larvae-L1-stage | 35 | 7.50 | 5.9e-19 |
| R02D3.7 | R02D3.7\_Larvae-L2-stage | 47 | 4.68 | 2.8e-18 |
| skn-1 | SKN-1\_Larvae-L3-stage | 44 | 5.02 | 5.5e-18 |
| F23B12.7 | F23B12.7\_Young-adult | 49 | 4.14 | 4.4e-17 |
| nhr-25 | NHR-25\_Larvae-L2-stage | 61 | 3.19 | 5.0e-17 |
| lsy-2 | LSY-2\_Larvae-L2-stage | 42 | 5.01 | 5.6e-17 |
| R02D3.7 | R02D3.7\_Larvae-L4-stage | 43 | 4.65 | 2.7e-16 |
| lsy-2 | LSY-2\_Embryos | 52 | 3.64 | 4.9e-16 |
| nhr-237 | NHR-237\_Embryos | 40 | 4.98 | 6.3e-16 |
| nhr-6 | NHR-6\_Larvae-L4-stage | 46 | 4.16 | 8.0e-16 |
| lin-13 | LIN-13\_Larvae-L2-stage | 51 | 3.66 | 1.0e-15 |
| pha-4 | PHA-4\_Young-adult | 41 | 4.69 | 1.7e-15 |
| pha-4 | PHA-4\_Larvae-L2-stage | 69 | 2.60 | 1.9e-15 |
| lin-35 | LIN-35\_Fed-L1-stage-larvae | 50 | 3.65 | 2.7e-15 |
| nhr-23 | NHR-23\_Larvae-L3-stage | 63 | 2.83 | 3.3e-15 |
| lin-15 | LIN-15B\_Fed-L1-stage-larvae | 44 | 4.18 | 4.4e-15 |
| nhr-77 | NHR-77\_Larvae-L3-stage | 43 | 4.24 | 7.0e-15 |
| dpl-1 | DPL-1\_Young-adult | 46 | 3.89 | 9.7e-15 |
| lsy-2 | LSY-2\_Larvae-L4-stage | 37 | 4.98 | 1.6e-14 |
| nhr-237 | NHR-237\_Larvae-L1-stage | 31 | 6.23 | 2.3e-14 |
| dve-1 | DVE-1\_Larvae-L4-stage | 48 | 3.57 | 3.9e-14 |
| F16B12.6 | F16B12.6\_Fed-L1-stage-larvae | 39 | 4.52 | 4.4e-14 |
| fos-1 | FOS-1\_Larvae-L3-stage | 46 | 3.64 | 1.1e-13 |
| fos-1 | FOS-1\_Fed-L1-stage-larvae | 56 | 2.95 | 1.2e-13 |
| C16A3.4 | C16A3.4\_Fed-L1-stage-larvae | 49 | 3.39 | 1.2e-13 |
| ceh-39 | CEH-39\_Embryos | 43 | 3.90 | 1.3e-13 |
| lin-13 | LIN-13\_Larvae-L4-stage | 42 | 3.99 | 1.4e-13 |
| F45C12.2 | F45C12.2\_Larvae-L2-stage | 32 | 5.53 | 1.7e-13 |
| lin-35 | LIN-35\_Starved-L1-stage-larvae | 36 | 4.76 | 1.7e-13 |
| dve-1 | DVE-1\_Late-Embryos | 52 | 3.15 | 1.9e-13 |
| ham-1 | HAM-1\_Fed-L1-stage-larvae | 62 | 2.63 | 2.3e-13 |
| jun-1 | JUN-1\_Larvae-L1-stage | 50 | 3.26 | 2.5e-13 |
| ceh-16 | CEH-16\_Larvae-L2-stage | 33 | 5.18 | 3.4e-13 |
| efl-1 | EFL-1\_Fed-L1-stage-larvae | 57 | 2.83 | 3.5e-13 |
| jun-1 | JUN-1\_Larvae-L3-stage | 44 | 3.69 | 3.7e-13 |
| gei-11 | GEI-11\_Larvae-L3-stage | 57 | 2.83 | 3.8e-13 |
| sax-3 | SAX-3\_Larvae-L4-stage | 62 | 2.60 | 4.1e-13 |
| pax-1 | PAX-1\_Embryos | 27 | 6.50 | 9.3e-13 |
| ceh-26 | CEH-26\_Late-Embryonic-stage | 40 | 3.98 | 9.5e-13 |
| aly-2 | ALY-2\_Larvae-L3-stage | 34 | 4.79 | 1.0e-12 |
| dpl-1 | DPL-1\_Fed-L1-stage-larvae | 54 | 2.90 | 1.2e-12 |
| jun-1 | JUN-1\_Larvae-L4-stage | 44 | 3.56 | 1.3e-12 |
| F45C12.2 | F45C12.2\_Fed-L1-stage-larvae | 52 | 2.97 | 2.0e-12 |
| nhr-77 | NHR-77\_Larvae-L2-stage | 33 | 4.85 | 2.0e-12 |
| lsy-2 | LSY-2\_Fed-L1-stage-larvae | 55 | 2.81 | 2.1e-12 |
| F45C12.2 | F45C12.2\_Larvae-L3-stage | 35 | 4.50 | 2.3e-12 |
| efl-1 | EFL-1\_Larvae-L1-stage | 57 | 2.71 | 2.3e-12 |
| ceh-38 | CEH-38\_Larvae-L3-stage | 52 | 2.88 | 6.6e-12 |
| nfya-1 | NFYA-1\_Larvae-L3-stage | 49 | 3.03 | 8.6e-12 |
| gei-11 | GEI-11\_Larvae-L2-stage | 47 | 3.15 | 9.1e-12 |
| gei-11 | GEI-11\_Fed-L1-stage-larvae | 54 | 2.76 | 9.3e-12 |
| hlh-30 | HLH-30\_Late-Embryos | 35 | 4.27 | 1.0e-11 |
| R02D3.7 | R02D3.7\_Larvae-L3-stage | 61 | 2.45 | 1.2e-11 |
| hpl-2 | HPL-2\_Fed-L1-stage-larvae | 62 | 2.42 | 1.2e-11 |
| nhr-11 | NHR-11\_Larvae-L2-stage | 32 | 4.63 | 1.8e-11 |
| zag-1 | ZAG-1\_Fed-L1-stage-larvae | 32 | 4.63 | 1.8e-11 |
| efl-1 | EFL-1\_Young-adult | 47 | 3.08 | 1.9e-11 |
| C34F6.9 | C34F6.9\_Larvae-L2-stage | 56 | 2.62 | 1.9e-11 |
| aha-1 | AHA-1\_Fed-L1-stage-larvae | 18 | 10.43 | 1.9e-11 |
| ham-1 | HAM-1\_Larvae-L4-stage | 62 | 2.36 | 3.6e-11 |
| nhr-129 | NHR-129\_Larvae-L2-stage | 69 | 2.14 | 4.2e-11 |
| nhr-76 | NHR-76\_Larvae-L4-stage | 40 | 3.52 | 4.6e-11 |
| unc-62 | UNC-62\_Day-Four-Young-Adult | 36 | 3.92 | 4.7e-11 |
| unc-62 | UNC-62\_Young-adult-Day-4 | 36 | 3.92 | 4.7e-11 |
| eor-1 | EOR-1\_Larvae-L3-stage | 56 | 2.55 | 5.7e-11 |
| egl-5 | EGL-5\_Larvae-L3-stage | 44 | 3.14 | 8.3e-11 |
| ztf-7 | ZTF-7\_Larvae-L4-stage | 40 | 3.43 | 1.0e-10 |
| sax-3 | SAX-3\_Larvae-L2-stage | 44 | 3.12 | 1.1e-10 |
| W03F9.2 | W03F9.2\_L4-Young-Adult-stage-larvae | 74 | 1.97 | 1.4e-10 |
| hlh-30 | HLH-30\_Larvae-L4-stage | 40 | 3.39 | 1.4e-10 |
| elt-1 | ELT-1\_Larvae-L3-stage | 31 | 4.35 | 2.1e-10 |
| zag-1 | ZAG-1\_Larvae-L2-stage | 41 | 3.26 | 2.1e-10 |
| gei-11 | GEI-11\_Young-adult | 35 | 3.81 | 2.3e-10 |
| fos-1 | FOS-1\_Larvae-L2-stage | 61 | 2.29 | 2.5e-10 |
| nfya-1 | NFYA-1\_Late-Embryos | 50 | 2.69 | 3.2e-10 |
| nhr-77 | NHR-77\_Larvae-L4-stage | 66 | 2.13 | 3.5e-10 |
| C01B12.2 | C01B12.2\_Larvae-L2-stage | 63 | 2.20 | 4.2e-10 |
| pes-1 | PES-1\_Larvae-L4-stage | 57 | 2.39 | 4.2e-10 |
| aha-1 | AHA-1\_Larvae-L4-stage | 26 | 5.16 | 4.4e-10 |
| dpl-1 | DPL-1\_Larvae-L4-stage | 62 | 2.23 | 4.9e-10 |
| elt-3 | ELT-3\_Embryos | 40 | 3.25 | 4.9e-10 |
| fos-1 | FOS-1\_Larvae-L4-stage | 28 | 4.70 | 5.1e-10 |
| nhr-77 | NHR-77\_Fed-L1-stage-larvae | 54 | 2.45 | 1.0e-09 |
| sem-4 | SEM-4\_Larvae-L2-stage | 52 | 2.52 | 1.1e-09 |
| unc-39 | UNC-39\_Embryos | 23 | 5.73 | 1.2e-09 |
| aly-2 | ALY-2\_Fed-L1-stage-larvae | 43 | 2.95 | 1.2e-09 |
| nhr-76 | NHR-76\_Larvae-L3-stage | 32 | 3.89 | 1.5e-09 |
| lsy-2 | LSY-2\_Larvae-L1-stage | 62 | 2.16 | 1.7e-09 |
| ztf-4 | ZTF-4\_Larvae-L3-stage | 23 | 5.44 | 3.0e-09 |
| nhr-2 | NHR-2\_Embryos | 29 | 4.17 | 3.1e-09 |
| unc-62 | UNC-62\_Fed-L1-stage-larvae | 20 | 6.40 | 4.2e-09 |
| pha-4 | PHA-4\_Larvae-L4-stage | 42 | 2.89 | 4.7e-09 |
| ztf-4 | ZTF-4\_Larvae-L1-stage | 20 | 6.32 | 5.1e-09 |
| nhr-28 | NHR-28\_Larvae-L4-stage | 63 | 2.08 | 5.4e-09 |
| zag-1 | ZAG-1\_Larvae-L3-stage | 25 | 4.75 | 6.2e-09 |
| nhr-6 | NHR-6\_Larvae-L2-stage | 48 | 2.53 | 9.0e-09 |
| ces-1 | CES-1\_Embryos | 51 | 2.41 | 9.0e-09 |
| ces-1 | CES-1\_Larvae-L4-stage | 25 | 4.61 | 1.2e-08 |
| mab-5 | MAB-5\_Larvae-L2-stage | 30 | 3.78 | 1.4e-08 |
| alr-1 | ALR-1\_Larvae-L2-stage | 46 | 2.58 | 1.4e-08 |
| ceh-38 | CEH-38\_Larvae-L4-stage | 33 | 3.43 | 1.6e-08 |
| mef-2 | MEF-2\_Fed-L1-stage-larvae | 20 | 5.70 | 2.8e-08 |
| nhr-21 | NHR-21\_Larvae-L2-stage | 22 | 5.01 | 3.7e-08 |
| nhr-237 | NHR-237\_Larvae-L2-stage | 14 | 9.25 | 3.8e-08 |
| ztf-4 | ZTF-4\_Larvae-L2-stage | 25 | 4.17 | 8.2e-08 |
| unc-62 | UNC-62\_Larvae-L3-stage | 33 | 3.15 | 1.2e-07 |
| zip-2 | ZIP-2\_Larvae-L4-stage | 18 | 5.90 | 1.3e-07 |
| nhr-28 | NHR-28\_Larvae-L3-stage | 18 | 5.79 | 1.7e-07 |
| zag-1 | ZAG-1\_Larvae-L4-stage | 26 | 3.79 | 2.4e-07 |
| sax-3 | SAX-3\_Larvae-L3-stage | 26 | 3.73 | 3.3e-07 |
| ces-1 | CES-1\_Fed-L1-stage-larvae | 24 | 4.01 | 3.7e-07 |
| ces-1 | CES-1\_Larvae-L3-stage | 23 | 3.96 | 9.9e-07 |
| lin-35 | LIN-35\_Young-adult | 28 | 3.22 | 1.6e-06 |
| gei-11 | GEI-11\_Embryos | 22 | 3.95 | 2.1e-06 |
| fkh-2 | FKH-2\_Larvae-L3-stage | 23 | 3.56 | 6.2e-06 |
| peb-1 | PEB-1\_Larvae-L2-stage | 18 | 4.23 | 1.5e-05 |
| daf-12 | DAF-12\_Larvae-L3-stage | 13 | 6.03 | 1.7e-05 |
| sax-3 | SAX-3\_Fed-L1-stage-larvae | 19 | 3.73 | 4.3e-05 |
| aly-2 | ALY-2\_Larvae-L2-stage | 15 | 4.66 | 4.7e-05 |
| nhr-12 | NHR-12\_Larvae-L2-stage | 8 | 9.29 | 1.5e-04 |
| ztf-11 | ZTF-11\_Embryos | 12 | 5.32 | 1.6e-04 |
| nhr-10 | NHR-10\_Larvae-L4-stage | 15 | 4.17 | 1.7e-04 |
| ztf-11 | ZTF-11\_Larvae-L3-stage | 10 | 6.17 | 3.0e-04 |
| mab-5 | MAB-5\_Embryos | 12 | 4.36 | 1.0e-03 |
| skn-1 | SKN-1\_Larvae-L4-stage | 7 | 8.17 | 1.2e-03 |
| ama-1 | AMA-1\_Larvae-L3-stage | 21 | 2.68 | 1.4e-03 |
| unc-62 | UNC-62\_Larvae-L2-stage | 16 | 3.27 | 1.4e-03 |
| med-1 | MED-1\_Embryos | 11 | 4.49 | 1.7e-03 |
| unc-39 | UNC-39\_Larvae-L2-stage | 11 | 4.43 | 1.8e-03 |
| sea-2 | SEA-2\_Larvae-L3-stage | 15 | 3.25 | 2.5e-03 |
| nhr-23 | NHR-23\_Larvae-L2-stage | 9 | 4.60 | 6.3e-03 |
| elt-1 | ELT-1\_Larvae-L2-stage | 7 | 5.85 | 8.1e-03 |
| nhr-116 | NHR-116\_Larvae-L2-stage | 10 | 3.80 | 1.2e-02 |
| nhr-237 | NHR-237\_Larvae-L3-stage | 3 | 22.27 | 1.2e-02 |
